# The phosphorylation of Hsp20 enhances its association with amyloid-β to increase protection against neuronal cell death

**DOI:** 10.1016/j.mcn.2014.05.002

**Published:** 2014-07

**Authors:** Ryan T. Cameron, Steven D. Quinn, Lynn S. Cairns, Ruth MacLeod, Ifor D.W. Samuel, Brian O. Smith, J. Carlos Penedo, George S. Baillie

**Affiliations:** a Institute of Cardiovascular and Medical Science, College of Veterinary, Medical and life sciences, University of Glasgow, Glasgow G128QQ, UK; b SUPA School of Physics and Astronomy, University of St Andrews, North Haugh, Fife KY169SS, UK

**Keywords:** Hsp20, Aβ oligomerisation, Peptide array

## Abstract

Up-regulation of Hsp20 protein levels in response to amyloid fibril formation is considered a key protective response against the onset of Alzheimer's disease (AD). Indeed, the physical interaction between Hsp20 and Aβ is known to prevent Aβ oligomerisation and protects neuronal cells from Aβ mediated toxicity, however, details of the molecular mechanism and regulatory cell signalling events behind this process have remained elusive. Using both conventional MTT end-point assays and novel real time measurement of cell impedance, we show that Hsp20 protects human neuroblastoma SH-SY5Y cells from the neurotoxic effects of Aβ. In an attempt to provide a mechanism for the neuroprotection afforded by Hsp20, we used peptide array, co-immunoprecipitation analysis and NMR techniques to map the interaction between Hsp20 and Aβ and report a binding mode where Hsp20 binds adjacent to the oligomerisation domain of Aβ, preventing aggregation. The Hsp20/Aβ interaction is enhanced by Hsp20 phosphorylation, which serves to increase association with low molecular weight Aβ species and decrease the effective concentration of Hsp20 required to disrupt the formation of amyloid oligomers. Finally, using a novel fluorescent assay for the real time evaluation of morphology-specific Aβ aggregation, we show that phospho-dependency of this effect is more pronounced for fibrils than for globular Aβ forms and that 25mers corresponding to the Hsp20 N-terminal can be used as Aβ aggregate inhibitors. Our report is the first to provide a molecular model for the Hsp20/Aβ complex and the first to suggest that modulation of the cAMP/cGMP pathways could be a novel route to enhance Hsp20-mediated attenuation of Aβ fibril neurotoxicity.

## Introduction

One of the pathological hallmarks of Alzheimer's disease (AD) is the extracellular disposition of amyloid-like filaments that form neuritic plaques in the brain. The principle component of amyloid plaques is a small peptide known as amyloid-β (Aβ), which is derived from sequential proteolytic cleavage of the amyloid precursor protein (APP) (Hardy and Selkoe, 2002). Increases in Aβ levels following an imbalance between the rates of production and clearance of the peptide, promote Aβ oligomerisation and lead to the formation of both insoluble fibrillar deposits and soluble Aβ oligomers. Both types of Aβ oligomers promote neuronal dysfunction and cell death leading to neurodegeneration (Harrison et al., 2007). This series of events is described as the “amyloid cascade hypothesis” and is supported by a wealth of biochemical and genetic data, though recent failures of a number of anti-Aβ aggregation drugs have cast some doubt on the hypothesis (Reitz, 2012). The most abundant peptide fragment found in AD is Aβ_1–40_, which accounts for approximately 90% of amyloid plaques, whereas the remaining 10% is made up of the more amyloidogenic fragment Aβ_1–42_. These short peptides are unstable and readily aggregate to form fibrils and a variety of other aggregated species that have been shown to be highly cytotoxic (Morgan et al., 2004).

Small Heat Shock Proteins (sHsps) are a group of ATP-independent chaperones that can prevent the aggregation of mis-folded proteins and as such, are protective against a number of protein aggregation diseases (Eyles and Gierasch, 2010). This is particularly evident in the field of neurological disease where sHsp proteins have been shown to have a protective role against Alzheimer's, Parkinson's and Huntington's diseases (reviewed in (Brownell et al., 2012)). One of the ten known sHsps, Hsp20, has been specifically linked with AD as it associates with pathological lesions in diseased brains (Wilhelmus et al., 2006a). These included senile plaques (SP) and cerebral amyloid angiopathies (CAA) both of which consist mainly of aggregated Aβ (Wilhelmus et al., 2009). Expression of Hsp20 has also been observed in reactive astrocytes and microglia found surrounding both SP and CAA (Wilhelmus et al., 2006a). The co-localisation of Hsp20 with Aβ aggregates within AD brain tissue suggests that Hsp20 may represent an endogenous neuronal protection mechanism to combat or prevent Aβ oligomerisation. Indeed, the physical interaction between Hsp20 and Aβ has been reported to prevent Aβ oligomerisation (Lee et al., 2006, Wilhelmus et al., 2006b) and protect model cell lines from Aβ mediated toxicity (Lee et al., 2006, Wilhelmus et al., 2006b), however, the molecular nature of this interaction has remained a mystery. Using peptide array technology (Frank, 2002), we have mapped the sites of interaction on both Hsp20 and Aβ and in doing so, can now shed light on the mechanism behind the unique ability of Hsp20 to regulate the aggregation of Aβ. We report that the PKA/PKG consensus site (RRAS) on Hsp20 is a key regulator of the chaperone's avidity for Aβ_1–42_ and directs association of the chaperone to structural elements of the peptide in order to prevent accumulation of neurotoxic Aβ species in SH-SY5Y cells. This data suggests that the cAMP/cGMP signalling pathway can “switch on” protection against Aβ-induced cell death and we propose that this novel signalling axis represents a therapeutic target for the reduction of Aβ associated neurodegeneration.

## Results

### Mapping the interaction between Hsp20 and Aβ_1–42_ using peptide array

**Fig. 1:**
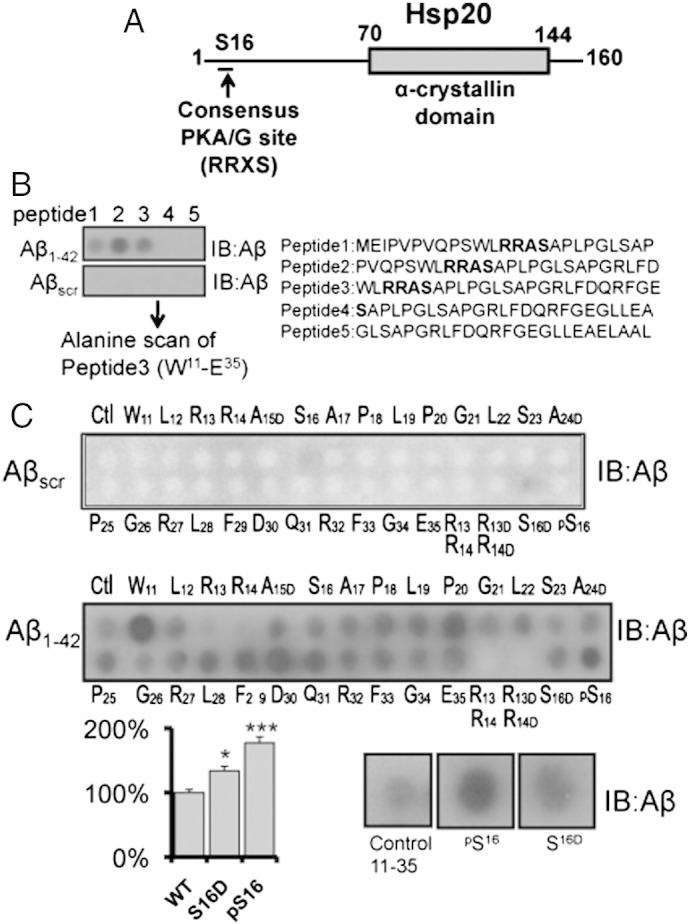
Mapping the interaction between Hsp20 and Aβ_1–42_. Peptide array was used to map the domains responsible for Hsp20/Aβ_1–42_ interaction. (A) Diagram of domain structure of Hsp20 highlighting the PKA/PKG site located in the N-terminal domain and the conserved α-crystallin domain located between residues 70 and 144. (B) Peptide array libraries of Hsp20 25mers were probed with either Aβ_1–42_ or Aβ_scr_. (C) Alanine scanning arrays of peptide 3 (W^11^–E^35^) were probed with either Aβ_1–42_ (middle panel) or Aβ_scr_ (upper panel) to determine the Hsp20 amino acids that are essential for Aβ_1–42_ binding. The association of Aβ_1–42_ with substitution arrays in which serine 16 was replaced by a phospho-serine or phospho-mimetic substitution (serine changed to aspartic acid) was also evaluated (lower panel). * = p < 0.05, ** = p < 0.01 using Student-*t*-test (n = 4).

As the interaction between Hsp20 and Aβ_1–42_ had previously been shown (Lee et al., 2006, Wilhelmus et al., 2006b), we decided to use synthetic peptide array technology to map the interaction domains between Hsp20 and Aβ_1–42._ We have recently used this technique to successfully characterize the molecular interactions that underpin two other protein complexes that include Hsp20; Hsp20–PDE4 (Sin et al., 2011) and Hsp20–AKAP Lbc (Edwards et al., 2012b). Peptide arrays of overlapping 25-mer peptides, sequentially shifted by 5 amino acids and spanning the entire sequence of Hsp20 (domain structure depicted in Fig. 1A) were incubated with Aβ_1–42_ or a scrambled version of Aβ_1–42_ (Aβ_scr_). The array was developed using antibodies against Aβ_1–42_ to identify the Hsp20 25mers that were able to capture Aβ_1–42_. Dark spots represent positive areas of Aβ_1–42_ interaction whereas clear spots are negative for the association (Fig. 1B). Whilst no signal was observed when the arrays were incubated with Aβ_scr_, positive signals were obtained for Hsp20 derived 25mer peptides 1,2 and 3 following incubation with Aβ_1–42_ (Fig. 1B). Peptides 1,2 and 3 span the amino acid sequence M^1^–E^35^ within the N-terminal domain of Hsp20 (Fig. 1A), which contains the PKA consensus site at serine 16 (Fan et al., 2004). pt?>To gain insight into which amino acids within Hsp20 might be critical in binding to Aβ_1–42_, we focused on the W^11^–E^35^ region of Hsp20 and using a ‘parent’ 25-mer peptide, generated 25 progeny of this peptide where each amino acid was sequentially mutated to alanine (or to aspartate if the residue is alanine) to provide an alanine-scanning array (Fig. 1C). The resulting library of peptides was probed with Aβ_1–42_ (Fig. 1C: middle panel) or Aβ_scr_ (Fig. 1C: upper panel). This identified a region of Hsp20 likely to be important for association with Aβ_1–42_, namely the double arginine (R^13^, R^14^) that forms part of the PKA consensus (RRASA). As this result suggested that the phosphorylation of serine 16 may influence the association of Hsp20 with Aβ_1–42_, we included either a phospho-serine 16 residue or a phospho-mimic substitution (S to D) (Fig. 1C, lower panels) into the Hsp20^11–35^ peptide. Significantly more Aβ_1–42_ bound to the 25mers that included the phospho-serine 16 residue or phospho-mimic substitution when compared to the native sequence.

**Fig. 2:**
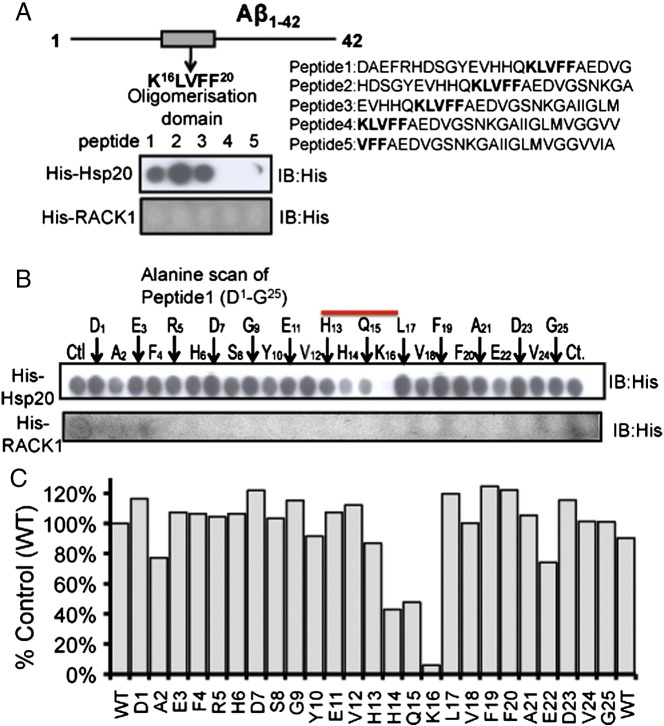
Mapping the interaction between Aβ_1–42_ and Hsp20. (A) Diagram of the of Aβ_1–42_ peptide with oligomerisation domain highlighted. Peptide array libraries of 25mers that spanned the Aβ_1–42_ sequence were probed with either His-Hsp20 or His-RACK1. (B) Alanine scanning arrays of Aβ_1–42_ peptide 1 (D^1^–G^25^) were probed with either His-Hsp20 (upper panel) or His-RACK1 (lower panel) to determine the Aβ_1–42_ amino acids that are essential for Hsp20 binding. (C) Quantifications of spot density of peptides in B (typical of n = 3).

In an attempt to determine the sites on Aβ_1–42_ that interact with Hsp20, we constructed peptide arrays of Aβ_1–42_ and overlaid these with purified His-tagged Hsp20 or as a control, purified His-tagged RACK1 (Fig. 2A), an unrelated scaffold protein. Strong association of Hsp20 (but not RACK1) to the first 3 spots of the Aβ_1–42_ array (representing amino acids 1–35) was observed. Alanine scanning analysis of the first 25 amino acids of Aβ_1–42_ (Fig. 2B) showed that the tri-peptide spanning H^14^,Q^15^ and K^16^ was critical for Hsp20 binding (Fig. 2C). Interestingly, this region abridges the K^16^LVFF^20^ oligomerisation domain of Aβ_1–42_. This region is known as the pathogenic aggregation site of the peptide and is essential for the formation of beta-sheets and amyloidogenicity (Hilbich et al., 1992, Tjernberg et al., 1996). Taken together (Fig. 1, Fig. 2), our peptide array data suggest a mechanism where phospho-Hsp20 binds avidly to Aβ_1–42_ and prevents self-association of the peptide.

### Hsp20—mediated prevention of cellular Aβ_1–42_ toxicity

**Fig. 3:**
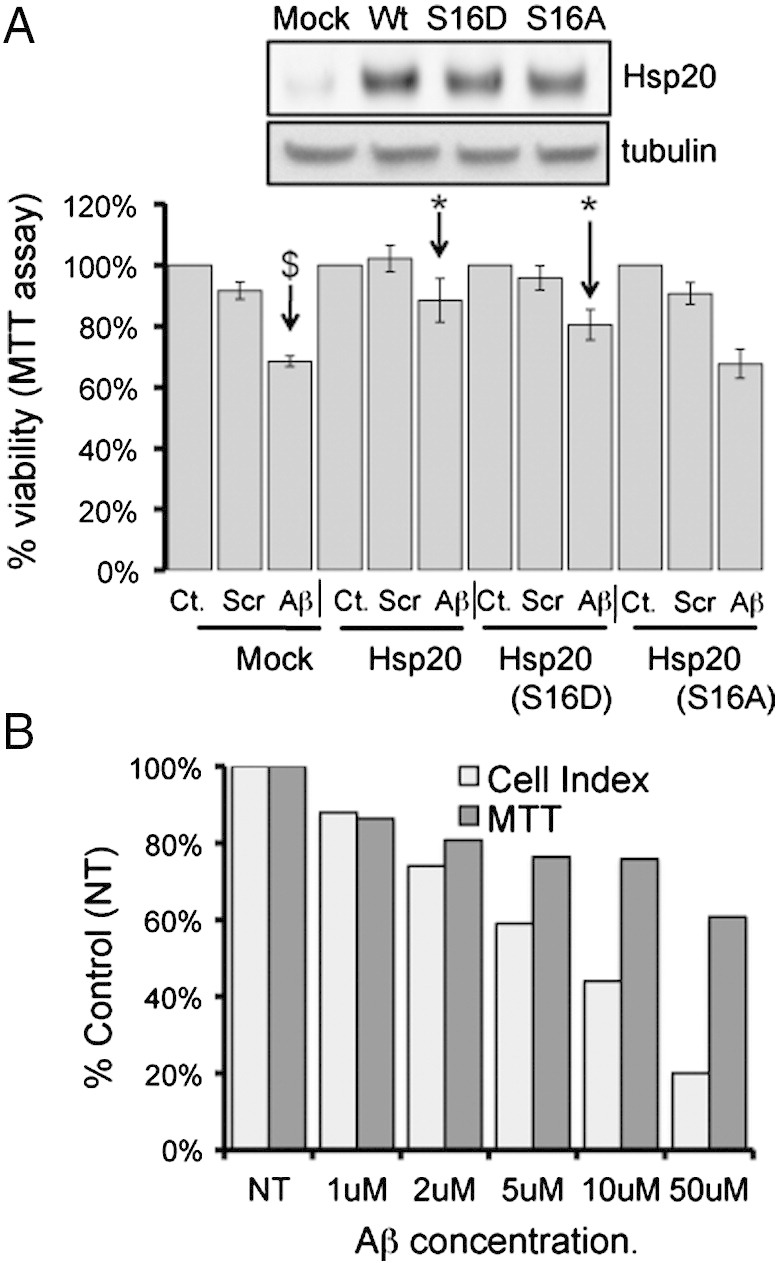
Cell viability assays to monitor Aβ_1–42_ mediated cytotoxicity. (A) The MTT cell viability assay was used to determine the effect of Aβ_1–42_ or Aβ_scr_ on cell viability of SH-SY5Y cells transfected with various constructs of Hsp20 (see inset for relative levels of expression). ($ = p < 0.001). Significant reduction in viability is denoted by * = p < 0.05 and $ = p < 0.001 using Student-*t*-test (n = 4). (B) Direct comparison of Aβ_1–42_ dose-dependent reduction in SH-SY5Y cell viability measured by MTT or using the xCELLigence real-time monitoring system. Data representative of n = 3.

**Fig. 4:**
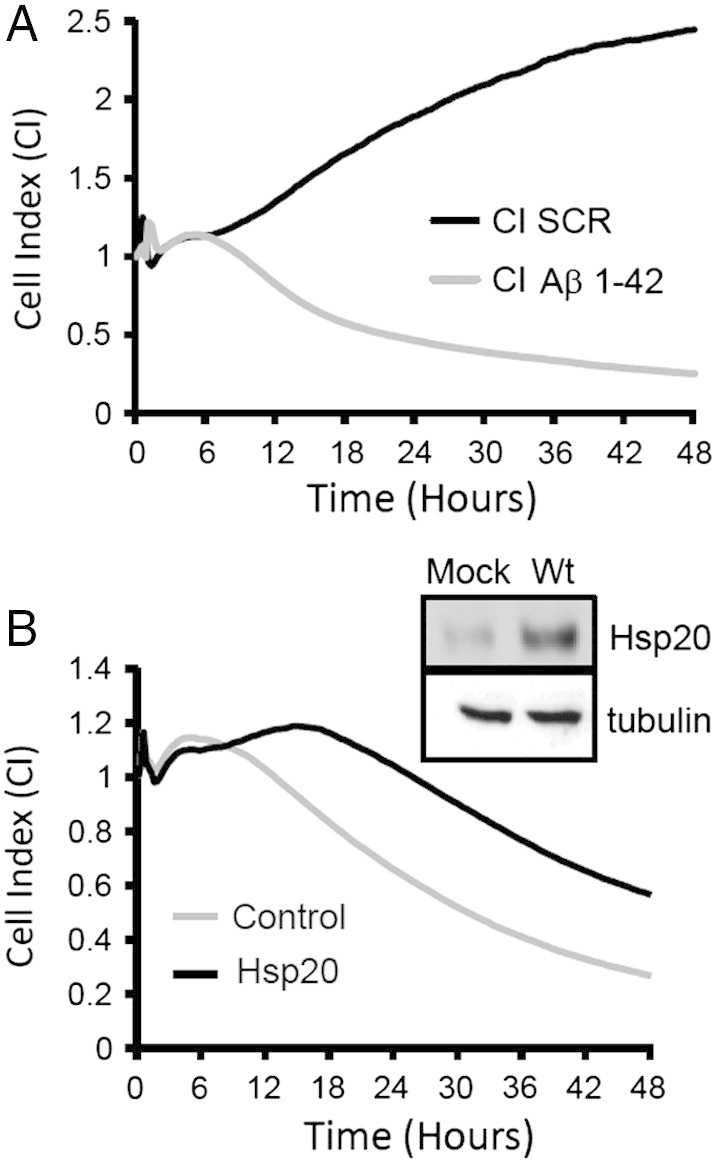
Real time monitoring of Aβ_1–42_ induced cytotoxicity in SH-SY5Y cells. (A) Impedance growth profiles of SH-SY5Y cells were measured over 48 h following treatment with Aβ_1–42_ or Aβ_scr_ .(B) Impedance growth profiles of SH-SY5Y cells transfected with empty vector or Hsp20 (see inset for relative expression levels) were measured over 48 h following treatment with Aβ_1–42_.

To determine whether the ability of Hsp20 to protect neuronal cells is enhanced following phosphorylation at serine 16, we set up an MTT viability assay using undifferentiated SH-SY5Y cells (Fig. 3A). As expected, addition of Aβ_1–42_ but not Aβ_scr_, resulted in a significant ($ = p < 0.001) reduction in cell viability when compared with vehicle only control (Fig. 3A). The Aβ_1–42_-mediated reduction in cell viability was significantly reduced in cells that had been transfected with Hsp20 wild type or the phospho-mimic Hsp20 (S^16^D), but not the phospho-null Hsp20 mutant (S^16^A). (* = p < 0.05: comparing Aβ_1–42_ treated, transfected cells with Aβ_1–42_ treated mock transfected cells) suggesting that phosphorylation enhanced Hsp20 protection against Aβ_1–42_. Although the MTT assay is the most common means of assessing Aβ_1–42_ toxicity in neuronal cells (Lee et al., 2006, Datki et al., 2003), the assay is limited by its sensitivity (Mozes et al., 2012), lack of ability to detect neuroprotective effects (Lobner, 2000) and by the fact that it is an endpoint assay that supplies limited information about the temporal nature of the Aβ_1–42_ toxic effect. Recently, the use of impedance recording as a sensitive, real time, non-invasive measurement of neuronal cell growth has become increasingly popular. This technique has been shown to be an accurate and reliable method by which to decipher the kinetics of cell death in neuronal cultures (Diemert et al. 2012, Mosse et al., 2008), something that cannot be achieved using discontinuous methods such as MTT. Briefly, neuronal cells are cultured in 96-well plates that have a network of micro-electrodes in the base and changes in adherence, proliferation or cell morphology can be distinguished by the impedance readout (Xiao et al., 2002a, Xiao et al., 2002b) which is measured in arbitrary units called “Cell Index”. Comparing the toxicity dose response of Aβ_1–42_ in SH-SY5Y neuroblastoma cells (Fig. 3B) it was apparent that impedance was a more sensitive readout of cell viability than MTT, especially at Aβ_1–42_ concentrations of 5 μM and above (Fig. 3B). Analysis of SH-SY5Y growth curves over 48 h showed that cells were unaffected by Aβ_scr_, and proliferated at a constant rate over the time period (Fig. 4A). Addition of Aβ_1–42_, however, resulted in normal growth for the first 6 h, followed by a constant reduction in the cell index (Fig. 4A) that is characteristic of cell death (Diemert et al., 2012). Transfection of HSP20 into SH-SY5Y cells delayed the toxic effect of Aβ_1–42_ and slowed the decrease in cell index (Fig. 4B).

**Fig. 5:**
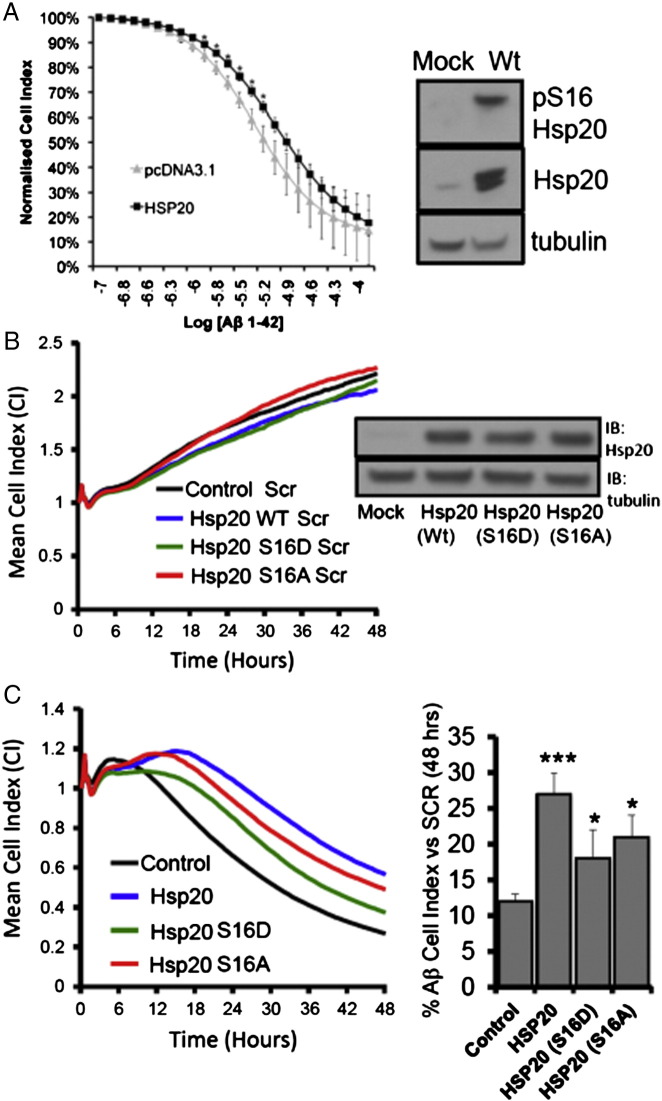
Hsp20 over-expression attenuates Aβ_1–42_ induced cytotoxicity in SH-SY5Y cells. (A) A dose response curve of cell viability (as measured by cell index) was constructed over a range of Aβ_1–42_ concentrations in SH-SY5Y cells transfected with empty vector or Hsp20. Relative levels of phospho-Hsp20 and total Hsp20 were determined by western blotting (left panels). Impedance growth profiles of SH-SY5Y cells transfected with empty vector, Hsp20Wt, Hsp20S16D, and Hsp20S16A (see inset for relative expression levels) were measured over 48 h following treatment with (B) Aβ_scr_ or (C) Aβ_1–42_. Quantifications of cell index at 48 h compared with the scrambled control in (A) were determined (n = 3) and statistical evaluation undertaken * = p < 0.05 and *** = p < 0.001 using Student-*t*-test.

When this experiment was repeated with increasing amounts of Aβ_1–42_, transfection of HSP20 caused a significant right shift of the cell index dose response curve (Fig. 5A left panel), again signifying that Hsp20 could protect against amyloid toxicity. It is noteworthy that the levels of phospho-HSP20, as well as exogenous HSP20, were elevated in these cells (Fig. 5A, right panel). To further investigate whether HSP20 phosphorylation had a bearing on Aβ_1–42_ toxicity, SH-SY5Y cells were transfected with HSP20 wild type, the phospho-mimic HSP20 (S^16^D), the phospho-null Hsp20 mutant (S^16^A) or vector alone (control) (Fig. 5B right panel). As expected, all transfected cells were unaffected by Aβ_scr_, and grew at a constant rate (Fig. 5B left panel). Transfection of all the HSP20 species, however, significantly increased cell index when compared to control after 48 h (Fig. 5C left and right panels) of Aβ_1–42_ treatment.

### Hsp20 phosphorylation alters the ability to affect morphology of Aβ aggregates

Using a novel assay for the evaluation of Aβ_1–42_ aggregate formation (Quinn et al., 2014), we tested whether the phosphorylation state of Hsp20 at serine 16 impacted the chaperone's ability to prevent the oligomerisation of Aβ_1–42_. The assay relies on fluorescence self-quenching between Aβ_1–42_ peptides labelled at the N-terminal position with HiLyte Fluor 555 (Aβ_555_). Basically, amyloid self-assembly brings the covalently attached fluorescence dyes into close proximity to induce a fluorescence quenching process that can be used to monitor the aggregation process in real time (Garai and Frieden, 2013). Here, we have used this method to investigate the inhibitory properties of Hsp20 against Aβ_1–42_ aggregation. We explored the fluorescence response of Aβ_555_ in the presence of Hsp20 at experimental conditions known to promote different morphologies. For instance, it has been shown that low concentrations of 1,1,1,3,3,3-hexafluoro-2-propanol (HFIP) (1–4% v/v) promote the formation of ring-like and globular structures, whilst fibril-like morphologies are formed in the presence of physiological (150 mM) concentrations of NaCl at 37 °C. We have observed a pronounced difference in the effect of wild type Hsp20 to the amyloid aggregation process under both experimental conditions suggesting a certain degree of morphology-specificity for the interaction of Hsp20 and amyloid aggregates.

**Fig. 6:**
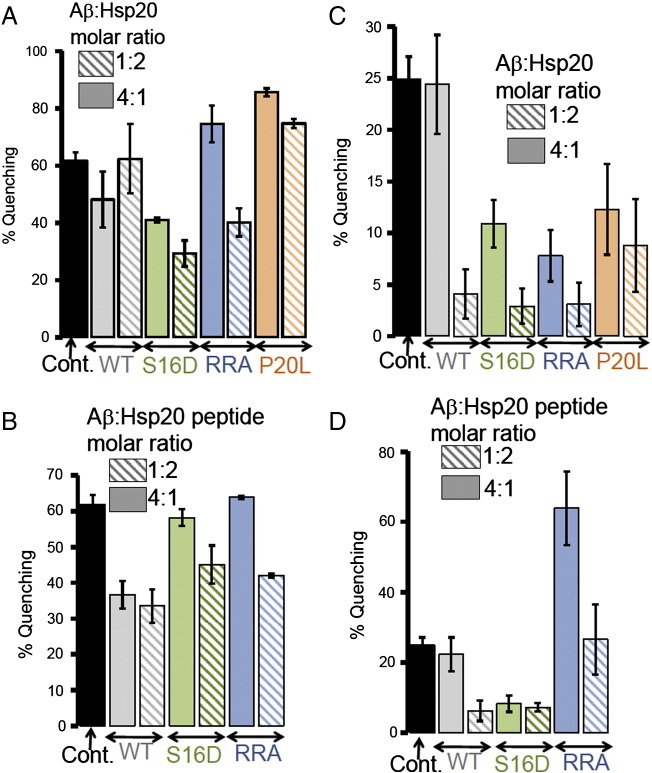
Evaluation of morphology-specific inhibition of Aβ_1–42_ aggregation by Hsp20 using a novel fluorescence self-quenching assay. The interaction between Hsp20 variants and Aβ_1–42_ labelled at the N-terminus with HiLyte Fluor 555 (Aβ_555_) was monitored using fluorescence self-quenching under globular (A) and fibrillar (C) growing conditions. The interaction between Hsp20 N-terminal 25mers and Aβ_1–42_ labelled at the N-terminus with HiLyte Fluor 555 (Aβ_555_) was monitored using fluorescence self-quenching under globular (B) and fibrillar (D) growing conditions. WT = wild type Hsp20, S16D = a phosphomimetic HSP20, RRA = a construct that is defective in binding Aβ_1–42_, and P20L = a polymorph (a naturally occurring mutant that is known to reduce the capacity of Hsp20 to be phosphorylated at serine 16).

In the absence of Hsp20, the addition of 1.5% of HFIP to a freshly prepared non-aggregated solution of 0.3 μM Aβ_555_ in 50 mM Tris-HCl buffer (pH 7.9) induced a 62 ± 3% decrease in fluorescence intensity (Fig. 6A) and 25 ± 2% under fibril-growing conditions (Fig. 6C). When the same experiments were repeated in the presence of Hsp20-WT, we observed a significant inhibition of amyloid growth under fibril-like conditions when using a molar excess of Hsp20-WT (i.e., 1:2 molar ratio Aβ:Hsp20), with the efficiency of the self-quenching process decreasing by 4-fold from 25 ± 2 to 4 ± 2% (Fig. 6B). In contrast, little inhibition was detected under HFIP-induced aggregation, or under any experimental conditions, when using a 4:1 molar excess of Aβ over Hsp20 (Fig. 6A and B). To get further insights into the mechanistic details of Hsp20 modulation of amyloid aggregation, we next repeated the fluorescence quenching assays using several relevant Hsp20 variants including S16D, RRA binding mutant and the P20L polymorph (a naturally occurring mutant that is known to affect its Hsp20 secondary structure and reduce its capacity to be phosphorylated at serine 16 (Nicolaou et al., 2008)).

The phospho-mimetic variant S16D exhibited higher inhibition efficiency (~ 50%) of globular- (Fig. 6A) and fibril-like (Fig. 6B) structures than the wild type, even at Aβ:S16D molar ratios (4:1) where Hsp20-WT showed no significant inhibitory effect. Interestingly, higher concentrations of S16D (1:2 Aβ:S16D molar ratio) had only a marginal effect in the fluorescence quenching accompanying the formation of globular structures (< 10% decrease in quenching). In contrast, the inhibition of fibrils was strongly increased as reflected by the relative decrease in fluorescence quenching from 11 ± 2% (4:1 Aβ:S16D molar ratio) to a practically undetectable level (3 ± 2%) when using a 1:2 molar ratio (Aβ:S16D) (Fig. 6B). In agreement with our peptide array data, these results confirm that replacing serine 16 with aspartic acid promotes the Hsp20/Aβ interaction and decreases the effective concentration of Hsp20 required to disrupt the formation of amyloid aggregates. This effect being more pronounced for fibrils than for globular-forming conditions. For the RRA and P20L variants the variation in fluorescence self-quenching showed also a remarkable dependence with the type of aggregate and the Aβ:Hsp20 variant molar ratio. P20L failed to inhibit the formation of globular structures at both molar ratios investigated (Fig. 6A). In fact, we observed a significant increase in fluorescence quenching from 62 ± 3% in the absence of P20L to values of 86 ± 1% and 75 ± 2% at 4:1 and 1:2 (Aβ:P20L) molar ratios, respectively. In contrast, P20L was able to inhibit the formation of fibrillar structures (Fig. 6B), although its efficiency at the highest molar ratio was lower (~ 9 ± 4% self-quenching efficiency) than for the RRA and S16D variants (~ 3%). For the RRA mutant, the behaviour under fibril-like forming conditions is parallel to that observed for S16D (Fig. 6B), whilst its ability to disrupt the formation of globules was slightly lower than for S16D at similar molar ratios (Fig. 6A).

We next performed similar experiments, but using 25-mer peptide analogues of Hsp20 sequences that include the Hsp20/Aβ interaction motifs identified from peptide array studies (Fig. 1, Fig. 2). We used Hsp20-WT, the S16D and RRA variants (Fig. 6C and D). The most significant differences between experiments using full length proteins and peptide analogues can be described as follows: i) the 25-mer S16D variant is approximately 2-fold less efficient in disrupting the formation of fibrils and globular structures than the full-length form and ii) whilst the full-length Hsp20 RRA mutant protein was capable of efficiently inhibiting the formation of fibrillar structures, the 25-mer version of the RRA variant was unable to do so at both molar ratios. Actually, we observed a very pronounced and reproducible increase in fluorescence self-quenching (~ 64 ± 10%) with the 25-mer RRA at 4:1 molar ratio compared to the control experiment in the absence of RRA (25 ± 2 5), indicative of higher levels of aggregation. When a 1:2 molar ratio of Aβ:Hsp20 was used, the fluorescence self-quenching returned to values similar to those obtained in the absence of RRA (Fig. 6D).

### Nuclear magnetic resonance spectroscopy to monitor Aβ_1–40_ aggregation

**Fig. 7:**
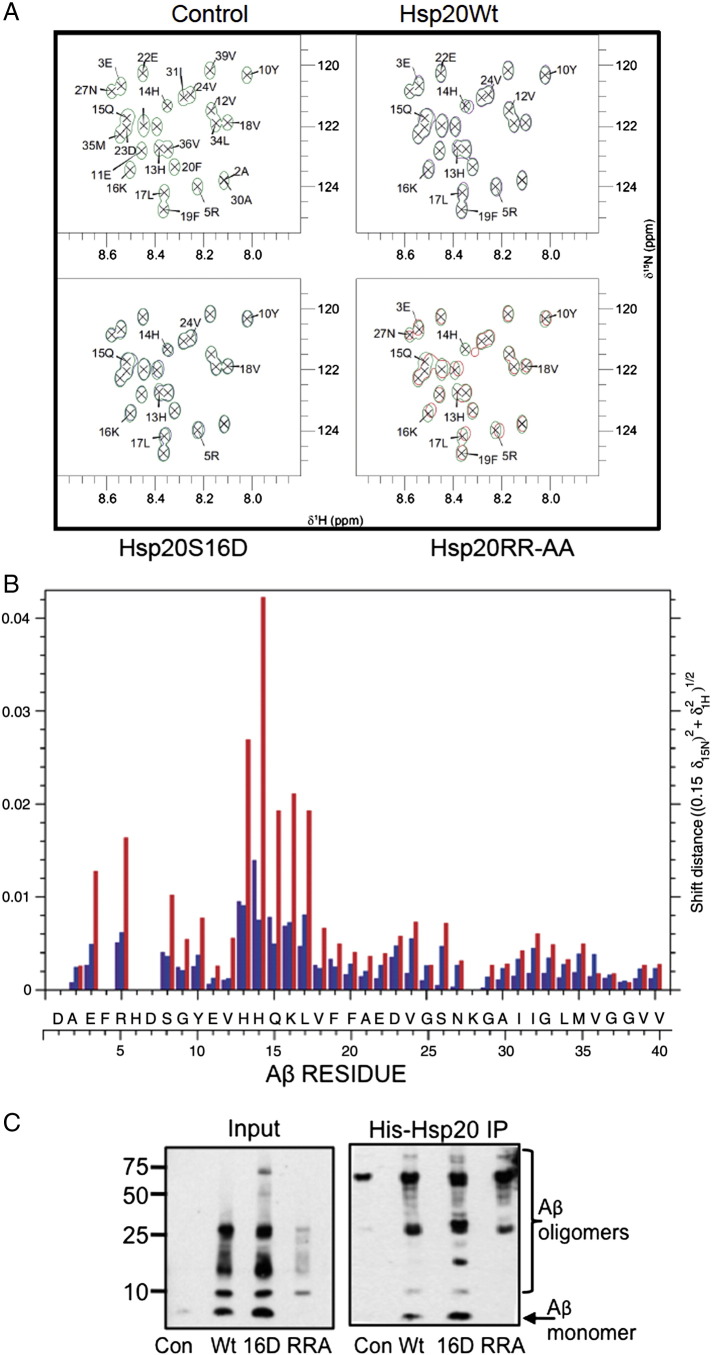
Chemical shift analysis of ^15^N-Aβ_1–40_ co-incubation with Hsp20. A. 2D HSQC experiment showing ^15^N-Aβ_1–40_ (green); co-incubated with either Hsp20 WT (blue), Hsp20-S16D (purple) or Hsp20-RAA (red) at 4 °C prior to aggregation. B. Chemical shift perturbation plot from same experiment as (A). Data plotted relative to the ^15^N-Aβ_1–40_ control. C. Hsp20 immunoprecipitations from the NMR samples were probed for Aβ following 4 day incubation under aggregating conditions. WT = wild type Hsp20, S16D = a phosphomimetic HSP20, RRA = a construct that is defective in binding Aβ_1–42_.

To support the data from the fluorescence self-quenching assay (Fig. 6) and peptide array experiments (Fig. 2), we undertook conventional NMR spectroscopic analysis to examine the effect of Hsp20 on the oligomerisation of synthetic ^15^N-labelled Aβ_1–40_ peptide. Small changes in chemical shifts were detectable across all residues compared to ^15^N-Aβ_1–40_ only control (Fig. 7A), but the largest changes are seen at the region proximal to the oligomerisation domain (KLVFF), spanning the sequence H^13^HQKL^17^, which includes the same region identified in the peptide array experiments (Fig. 7B). Hsp20-RRA induced the largest changes in chemical shift for residues within this region, whilst Hsp20-S16D increased the shift distance across 80% of the assigned residues relative Hsp20-WT. Following initial 1D ^1^H NMR and 2D ^15^N-HSQC spectral analysis, each sample was incubated in conditions that promote oligomerisation of ^15^N-Aβ_1–40_. Samples were then re-analysed in order to determine how much ^15^N-Aβ_1–40_ peptide would still be visible in solution given that aggregated species larger than 50 kDa are not detected using NMR spectroscopy (Kwan et al., 2011). As expected the ^15^N-Aβ_1–40_ only control had significantly reduced peak intensities suggesting reduced concentration of monomeric Aβ peptide via increased aggregation (compare Suppl Fig. 1A and Suppl Fig. 1B). The same was also true for the ^15^N-Aβ_1–40_ peptide co-incubated with the binding mutant Hsp20-RRA although to a lesser extent. However, both the Hsp20-WT and the Hsp20-S16D co-incubations maintained significantly more ^15^N-Aβ_1–40_ in its monomeric form when compared to both ^15^N-Aβ_1–40_ control and Hsp20-RRA (Suppl. Fig. 1A and B). In order to confirm that any loss in signal was the result of Aβ_1–40_ aggregation and not proteolytic degradation, the NMR samples were blotted for Aβ (Fig. 7C left panel). As expected, the levels of monomeric Aβ_1–40_ had virtually disappeared in the ^15^N-Aβ_1–40_ only control sample (lane 1) whereas monomeric and low molecular weight species were most prominent in Aβ samples that had been incubated with His-Hsp20-S16D (lane 3) followed by those that had been incubated with His-Hsp20-WT. In contrast, Aβ samples that contained His-Hsp20-RRA, exhibited no detectable levels of monomeric Aβ_1–40_ in solution and greatly reduced levels of low molecular weight species between 10 and 25 kDa (lane 4). To determine the nature of the Aβ_1–40_ aggregates that associates with Hsp20 under the conditions used for NMR studies, immunoprecipitates of Hsp20 were probed with an antibody against Aβ (Fig. 7C, right panel). In agreement with the notion that phosphorylation of Hsp20 at serine 16 increases the association of the chaperone with Aβ, Hsp20-S16D was able to pull-down more monomeric Aβ_1–40_ than the WT variant (lane 2 vs lane 3). Interestingly, Hsp20-S16D was also able to coIP an Aβ_1–40_ species around the size expected for Aβ tetramers (16 kDa) (Fig. 7C, right panel). This Aβ species was not detected in the Hsp20-WT IP despite there being species of this size in solution with Hsp20-WT post aggregation (Fig. 7C left panel). Despite similar levels of Hsp20-RRA precipitating with the His-agarose beads (data not shown), there was no low molecular weight Aβ_1–40_ species detected in the Hsp20 IP (Fig. 7C, right panel, lane 4).

Taken together, the data in Fig. 7 suggests that Hsp20 interacts with Aβ_1–40_ and prevents it from aggregating into higher molecular weight oligomers, even at a molar ratios of 1:4 (Hsp20:Aβ). Both Hsp20-WT and -S16D maintained significantly more LMW species of Aβ_1–40_ in solution than the Aβ_1–40_ only control. The interaction between all Hsp20 variants and Aβ_1–40_ was strongest at domains important for beta-sheet formation and oligomerisation of Aβ. Finally, the introduction of the phospho-mimetic S16D increased the chemical shifts at a number of residues and maintained the Aβ_1–40_ peptide in its non-toxic, random coil conformation more so than Hsp20-WT. These data validate the findings from the array data that suggest the phosphorylation of Hsp20 enhances its interaction with Aβ to inhibit amyloidogenesis.

## Discussion

Small heat-shock proteins have been shown for some time to have the capacity to bind Aβ peptides and inhibit aggregation and subsequent cytotoxicity in vitro (Kudva et al., 1997, Lee et al., 2006). In particular, Hsp20 has been shown to interact with soluble Aβ and inhibit its aggregation and routes of enhancing the interaction between sHSPs and Aβ has been identified as a potential therapeutic strategy (Wilhelmus et al., 2006b). In this report, we have for the first time, discovered a molecular mechanism by which the interaction between Aβ and Hsp20 may be regulated in SH-SY5Y neuroblastoma cells. Importantly, the phosphorylation of Hsp20 at serine 16 by PKA/G is known to induce the protective abilities of Hsp20 in a number of physiological processes associated with diseases of the heart (Edwards et al., 2012a, Fan and Kranias, 2011), however, the data presented here also describes a novel neuroprotective role for Hsp20 phosphorylation. In short, PKA/G phosphorylation of the chaperone enhances its association with Aβ (Fig. 1, Fig. 7C) on a motif that is proximal to the Aβ oligomerisation domain K^16^L^17^V^18^F^19^F^20^ (Fig. 2, Fig. 7A, B), which is necessary for the assembly of toxic aggregates (Beyreuther et al., 1992, Watanabe et al., 2001). This action serves to reduce the formation of higher order Aβ oligomers (Fig. 6, Fig. 7C), by inhibiting Aβ aggregation directly at the site of oligomerisation, to protect neuroblastoma cells from the cytotoxic effects of Aβ (Fig. 3, Fig. 4, Fig. 5). We propose that the positively charged residue of the Aβ oligomerisation domain, K^16^ (the only residue in the sequence essential for mediating binding of Hsp20, Fig. 2) forms a charge interaction with Hsp20 following the introduction of a negatively charged phosphate group at serine 16. It is noteworthy that the Aβ residue, K^16^, plays an important role in the non-amyloidogenic processing of APP, as α-secretase cleavage at this site does not generate the Aβ peptide (Zheng and Koo, 2006). The scanning array (Fig. 2) and NMR analysis (Fig. 7) also demonstrated that H^14^ and Q^15^ are important residues that mediate the binding of Hsp20. H^14^ has been shown to play an important role in the co-ordination of metal ion binding, such as zinc, and copper (Diaz et al., 2006). Such metal ions can have significant effect on aggregation propensity of Aβ (Olofsson et al., 2009). Interestingly, we also saw a reduction in binding when the Aβ glutamic acid residue (E^22^) was substituted for alanine (Fig. 2). Mutation of this residue causes severe early onset familial AD (Selkoe and Podlisny, 2002) resulting from an increased capacity to form fibrils when compared with wild type Aβ (Inayathullah and Teplow, 2011). This is particularly true of the ‘Dutch’ mutation (E22Q) (Levy et al., 1990) and the ‘Arctic’ mutation (E693G) (Nilsberth et al., 2001). It is possible that sub-optimal binding of Hsp20 to Aβ because of such mutations decreases the neuro-protective capacity of Hsp20 and promotes the onset of AD.

Early structural characterisation of Aβ_1–40_ in solution using NMR spectroscopy has shown that when in solution Aβ_1–40_ contains two helical regions spanning Q^15^–D^23^ and I^31^–M^35^, with the rest of the peptide adopting a random coil formation (Sticht et al., 1995). Our initial analysis of the chemical shift perturbations for all Hsp20 variants was most pronounced in these two helical regions and more importantly, we demonstrated that the introduction of the phospho-mimetic substitution (S16D) increased the shift difference in the large majority of residues relative to Hsp20-WT, suggesting that the introduction of a negative charge at serine 16 increases the interaction of Hsp20 with Aβ_1–40_. Greater shift differences between Hsp20-WT and -S16D were also detected in the region spanning residues G^29^–V^36^ which span the second helical region and suggest that phosphorylation of Hsp20 enhances its interaction with the both helical regions within Aβ_1–40_ in order to maintain it in its soluble conformation. Crucially, both of these regions interact with each other upon structural conversion into insoluble fibrils and current models show that the two regions fold into a β-strand-turn-β-strand conversion. This step is the primary nucleation event of β-sheet secondary structure, which is essential for fibrillar growth (Ahmed et al., 2010).

Rather unexpectedly, we found that the RRA ‘binding mutant’ induced the most pronounced changes in shift distance across all residues within Aβ_1–40_. This was most pronounced at the oligomerisation domain, particularly at residues H^13^ and H^14^ and is likely due to the removal of the two adjacent, positively charged arginine residues, removing the charge repulsion that would normally occur at the two histidine residues. Despite the Hsp20-RRA mutant inducing the biggest change in chemical shifts, this did not translate into increased aggregation inhibition, relative to Hsp20-WT and -S16D. Both Hsp20-WT and S16D maintained significant amounts of Aβ_1–40_ in solution in its monomeric conformation despite incubation under conditions that promote Aβ aggregation. The conformational transition of Aβ from random coil to α-helix to β-sheet structures is a key step in promoting neurotoxicity of the peptide (Simmons et al., 1994), therefore it appears that chaperone activity of Hsp20 functions to stabilise Aβ in a non-toxic conformation. Additionally, analysis of the in vitro pull-down assay with Hsp20-S16D and Aβ_1–40_ following aggregation (Fig. 7C), revealed distinct low molecular weight species at 17 kDa and 27 kDa that have previously been described as being neurotoxic (Lambert et al., 1998). This suggests that phospho-Hsp20 has a higher propensity to bind soluble toxic species relative to WT, a finding that was in agreement with data from a novel Aβ aggregation assay, where both full length Hsp20 protein and 25mer peptides spanning the N-terminal of Hsp20 and containing the S16D mutation were able to inhibit fibrillar growth. Interestingly, transducible phospho-mimetics based on the N-terminal sequence of Hsp20 have been developed, to combat a number of disease conditions including, keloid scarring, subarachnoid haemorrhage, and platelet aggregation (Edwards et al., 2011). Whether such peptides would have physiological efficacy in reducing fibril formation may be worthy of further investigation.

In summation, we present a novel, regulatory mechanism by which Hsp20 attenuates Aβ_1–42_ cytotoxicity by increasing its ability to inhibit two morphology distinct Aβ aggregation pathways relevant to physiological amyloidogenesis and early nucleation events. Hsp20 binds directly to domains involved in the structural conversion to neurotoxic Aβ species and functions as a chaperone to maintain Aβ in a soluble non-toxic conformation. Phospho-mimetic Hsp20 also binds to higher order structures which may represent a mechanism of solubilising hydrophobic Aβ_1–42_ conformations to neutralise toxicity or increase Aβ peptide clearance. Finally using a novel label-free cell monitoring system we were able to confirm that increased intracellular levels of phospho-Hsp20 protects against cytotoxicity in SH-SY5Y neuroblastoma cells associated with diffusible Aβ and that this protection is likely mediated through a direct interaction as opposed to the anti-apoptotic properties of Hsp20. Therefore, we believe that the PKA/G induced phosphorylation of Hsp20 represents a novel endogenous protection mechanism that may be targeted therapeutically for the treatment of AD.

## Experimental methods

### Aβ peptides

For cell-based assays synthetic Aβ peptides were purchased from rPeptide® (Georgia, USA). Aβ_1–42_ (A-1002) peptides are the recombinant form of the human Aβ peptide. Aβ_1–42_ scrambled peptide (Aβ_scr_) (A-1004) which is a rearranged version of the peptide that carries the overall weight and charge of Aβ_1–42_, was used as a control. Peptides were dissolved in DMSO at a concentration of 5 mg/ml and sonicated in a water bath for 15 min. Samples were aliquoted and stored at − 20 °C until required. To create neurotoxic Aβ_1–42_ derivatives the method of Lambert et al. was used (Lambert et al., 1998), where Aβ_1–42_ (or scrambled) peptides were brought to 100 μM in cold PBS and incubated at 4–8 °C for 24 h. The resulting aggregated peptides were added directly to cell culture medium typically at 1:10 dilution (Aβ:media). Samples from each 100 μM stock were taken for SDS-PAGE and western blotting analysis.

For NMR assays ^15^N uniformly labelled Aβ_1–40_ (A-1101-2) was also purchased from rPeptide® (Georgia, USA). In order to fully monomerise the peptide it was resuspended in 1% NH_4_OH and sonicated in a water bath for 15 min. The peptide concentration was brought to 400 μM with cold NMR buffer (50 mM NaPi (Na_2_HPO_4_) pH 7.5). The peptide was then dialysed in 4 l of cold NaPi for 2 h to remove NH_4_OH and then added directly to Hsp20 containing NaPi buffer for immediate analysis. Aβ_1–40_ was maintained below 4 °C in order to reduce aggregation.

For real-time Aβ_1–42_ aggregation assays synthetic Aβ_1–42_ peptides were purchased from Anaspec Inc. (USA), suspended in 100% 1,1,1,3,3,3-hexafluoro-2-propanol (HFIP) at 5 mg/ml and incubated for complete solubilisation at room temperature for 1.5 h. HFIP was subsequently removed by evaporation under vacuum for 4 h and stored at − 20 °C.

### Antibodies

The following antibodies were used in western blotting analysis: anti-Aβ_1–42_— Sigma-Aldrich (A8354), anti-Hsp20 — Upstate (07–490), anti-phospho-S16 Hsp20 — Abcam (ab58522), and alpha tubulin HRP — Abcam (ab40742). Secondary antibodies used: anti-mouse HRP — GE Healthcare (NXA931)and anti-rabbit — Sigma-Aldrich (A6154). For co-IPs: anti-Polyhistidine-agarose — Sigma-Aldrich (A5713).

### His-Hsp20 purification

The full length Hsp20 sequence was cloned into a pET28c vector (Novagen) in order to express an N-terminal His-tag and then transformed into competent BL21 cells (Invitrogen, Paisley). Cells were grown until OD_600_ ~ 1, 1 M of IPTG was then added and cells were grown for a further 24 h at 37 °C. The protein was then purified using nickel affinity chromatography. The resulting protein product was then checked for impurities on a 4–12% gel and then verified through western blotting techniques. Subsequent site-directed mutagenesis of this vector was carried out using Quikchange (Stratech) in accordance with manufacturer's protocol.

### SDS-PAGE & western blotting

SDS-PAGE analysis was done on NuPage® pre-cast gels in Invitrogen X-cell apparatus (Invitrogen, Paisley) using Laemmli 2 × loading buffer with 5% β-mercaptoethanol. MES-SDS running buffer was used, due to the low molecular weight of proteins involved. For western blotting (WB) analysis, samples were transferred using NuPage® X-cell blotting module onto a nitrocellulose membrane. Membranes were blocked using 5% milk in 1 × TBST (w/v). Antibodies were incubated in 1% milk in 1 × TBST (w/v) for either 1 h at room temperature or overnight at 4 °C. Signals were detected using enhanced chemiluminescence (ECL) systems and developed on an X-omat film developer.

### Peptide array

The Hsp20 protein sequence was split into overlapping 25 amino acid fragments that advanced from the N-terminal to the C-terminal in increments of 5 residues until the full length of Hsp20 was covered. Two copies of these Hsp20 25mer libraries were SPOT synthesized (Frank, 2002) on continuous cellulose membranes using Fmoc-chemistry with the Autospot-Robot ASS 222 (Intavis Bioanalytical Instruments AG, Köln, Germany). For the alanine scanning arrays, versions of Hsp20 25mer (residues 11–36) were synthesised to incorporate alanine residues in place of the endogenous amino acids and were progressively substituted from the N-terminal to C-terminal. In the event of alanine being the original residue an aspartic acid was incorporated. Additionally, two spots in Hsp20^11–36^ modified to incorporate either a phospho-serine or a phospho-mimic (aspartic acid) at the Hsp20 phosphorylation site (serine 16). Prior to use, the cellulose membrane was activated using analytical ethanol and then blocked with 5% milk/TBST (w/v) for 1 h. The Hsp20 arrays were then overlaid with either Aβ_1–42_ or Aβ_scr_ overnight at 4 °C. The arrays were then analysed using WB techniques. Analogous methods were used to probe overlapping Aβ_1–42_ with His-tagged HSP20 in order to determine which domains within Aβ_1–42_ are responsible for binding.

### Cell culture

Undifferentiated SH-SY5Y cells were grown in Dulbecco's Modified Eagle's Medium (DMEM) and F12-Ham's at a 1:1 ratio, media were supplemented with 10% (v/v) foetal bovine serum (FBS), 1% (v/v) l-GLUTAMINE, 1% (v/v) Minimum Essential Medium — with non-essential amino acids (MEM-NAA) and 1% (v/v) Pen/strep. Cells were cultured in a humidified, 5% (v/v) CO_2_, 37 °C incubator.

### Aβ toxicity assays

Full-length Hsp20 was cloned into pcDNA3.1/V5-His-TOPO vector (Invitrogen) and related mutants created using Quikchange (Stratech). The various Hsp20 constructs and an empty vector control were electroporated into SH-SY5Y cells using nucleofection kit V (Amaxa) in accordance with manufacturer's instructions. Cell were seeded at a density of 5 × 10^3^/well into seeded into 96-well plate for MTT-based assays or 96-well E-plate for xCELLigence based assays and left overnight to allow for cell re-attachment. Remaining cells were seeded into 6 well plates and harvested after 48 h to confirm expression of the various Hsp20 constructs. Addition of Aβ peptides and vehicle controls was carried out once cell index reached 1. The xCELLigence SP system (Acea) was used for real-time monitoring of cell growth for a minimum of 48 h post addition of Aβ peptides. The resulting data was analysed using (RTCA) real-time cell analyzer software (Roche) and exported to Excel. MTT based cell viability was carried out in parallel in accordance with Promega CellTiter 96® non-radioactive cell proliferation assay (G4000) in accordance with manufacturer's protocols and added 48 h post addition of Aβ peptides.

### Nuclear magnetic resonance spectroscopy

^15^N-labelled Aβ_1–40_ samples were combined with 1 mg/ml of various His-Hsp20 constructs to give a final concentration of 200μM of Aβ_1–40_ and 25 μM of Hsp20 (4:1 molar ratio) in 50 mM NaPi buffer, 200 μM Aβ_1–40_ only was used as a control.

NMR spectra were recorded on Bruker AVANCE 600 MHz spectrometer at 4 °C to assess pre-aggregation spectra prior to incubating all samples at 37 °C for 4 days under agitating conditions (300 rpm). Samples were then reanalysed at 4 °C to ascertain how much Aβ_1–40_ peptide remained in a solution. Following NMR analysis samples were centrifuged at 13000 rpm in order to remove insoluble aggregates that had formed during the aggregation process and supernatant was analysed using SDS-PAGE and western blotting to ensure any loss of signal was not due to proteolytic degradation of the ^15^N-labelled Aβ_1–40_ peptide.

Supernatants from each sample were then used to undertake co-immunopurification using anti-polyhistidine-agarose conjugated beads (Sigma-Aldrich, UK). 20 μl of His-agarose beads was added to 500 μl of the Aβ_1–40_:Hsp20 solutions and incubated at 4 °C overnight on a rotating wheel. Each sample was then spun at 6000 rpm to isolate the beads. Following the removal of supernatant beads were subjected to a further 3 washes in PBS prior to addition of 2 × SDS sample buffer. Samples were then run on an SDS-PAGE gel to verify the interaction between Aβ_1–40_ and Hsp20.

### Real-time Aβ aggregation protocol

The real-time aggregation has recently been described by Quinn et al. (in press).

### Statistical analysis

Data is expressed as the means ± SEM. Two group comparisons were evaluated using two-tailed Student's *t*-test. Differences were considered statistically significant when p-value was < 0.05.

## Appendix A Supplementary data

Supplementary figure. NMR Analysis of 15N-Aβ1-40 incubated with Hsp20 post aggregation. Suppl. Fig 1A Partial [1H,15N]-HSQC spectra of recombinant 15N-Aβ1-40 peptide (200μM) in 50mM sodium phosphate buffer. 15N-Aβ1-40 peptide only (green), 15N-Aβ1-40 and his-Hsp20 (50μM) (blue), 15NAβ1-40 and his-Hsp20-S16D (50μM) (purple), 15N-Aβ1-40 peptide and his-Hsp20-RRA (50μM) (red).Suppl. Fig 1B samples were re-analysed after 4 days of incubation under aggregating conditions.
