# Supplementary material for: The phosphorylation of Hsp20 enhances its association with amyloid-β to increase protection against neuronal cell death
Source: Mol Cell Neurosci. 2014 Jul;61:46–55. doi: 10.1016/j.mcn.2014.05.002 (PMC4148482; doi:10.1016/j.mcn.2014.05.002)
Supplement: Supplementary figure. — NMR Analysis of 15N-Aβ1-40 incubated with Hsp20 post aggregation. Suppl. Fig 1A Partial [1H,15N]-HSQC spectra of recombinant 15N-Aβ1-40 peptide (200μM) in 50mM sodium phosphate buffer. 15N-Aβ1-40 peptide only (green), 15N-Aβ1-40 and his-Hsp20 (50μM) (blue), 15NAβ1-40 and his-Hsp20-S16D (50μM) (purple), 15N-Aβ1-40 peptide and his-Hsp20-RRA (50μM) (red).Suppl. Fig 1B samples were re-analysed after 4 days of incubation under aggregating conditions. [file mmc1.pptx]

## Slide 1
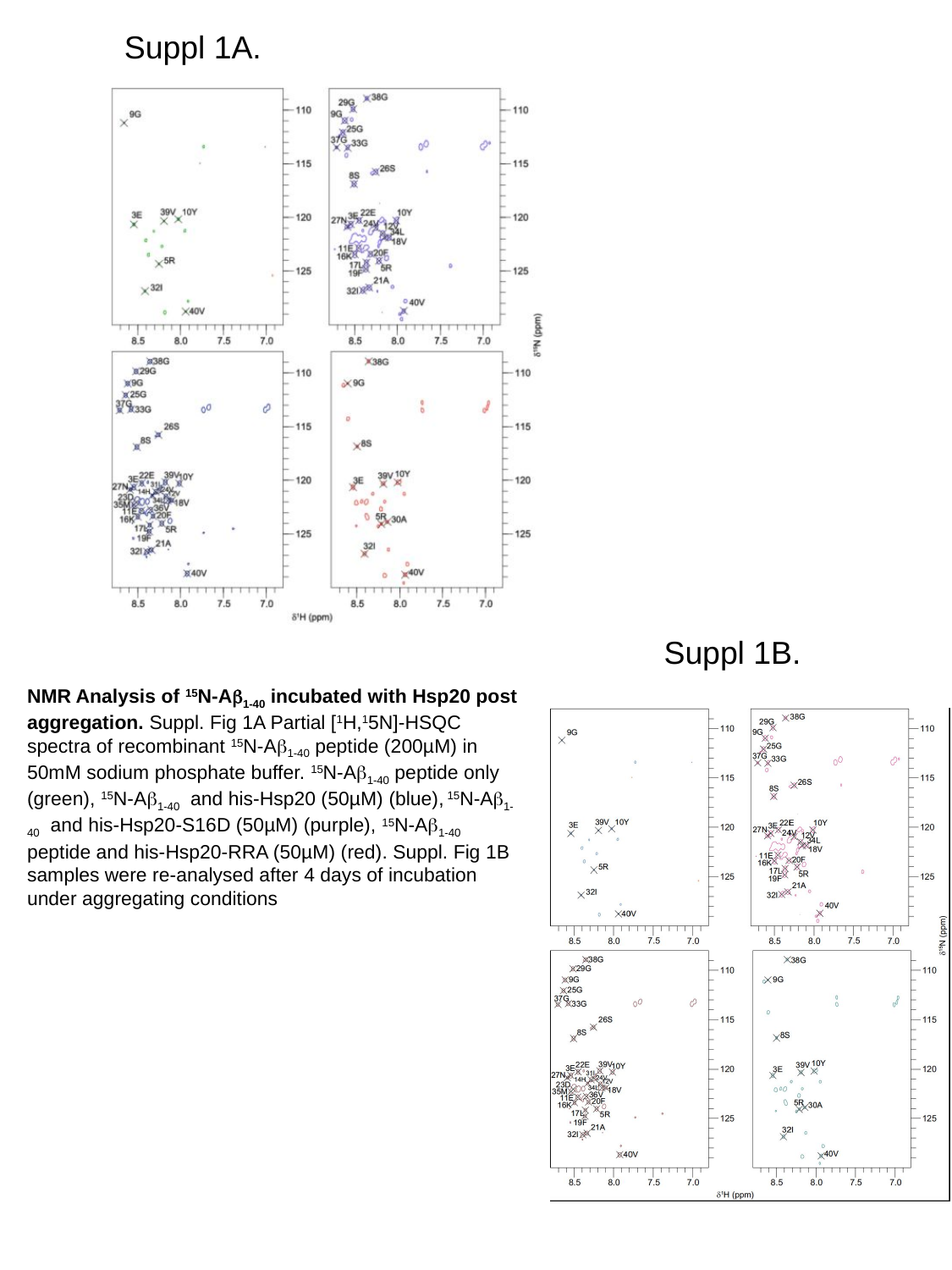

Suppl 1A.
Suppl 1B.
NMR Analysis of 15N-Ab1-40 incubated with Hsp20 post aggregation. Suppl. Fig 1A Partial [1H,15N]-HSQC spectra of recombinant 15N-Ab1-40 peptide (200µM) in 50mM sodium phosphate buffer. 15N-Ab1-40 peptide only (green), 15N-Ab1-40 and his-Hsp20 (50µM) (blue), 15N-Ab1-40 and his-Hsp20-S16D (50µM) (purple), 15N-Ab1-40 peptide and his-Hsp20-RRA (50µM) (red). Suppl. Fig 1B samples were re-analysed after 4 days of incubation under aggregating conditions
